# The Province of the Dentist

**Published:** 1860-12

**Authors:** J. Taft


DENTAL REGISTER OF THE WEST.

VOL. XIV.] DECEMBER, 1860. [NO. 6.

### Original Essays and Communications.

### THE PROVINCE OF THE DENTIST.

BY J. TAFT.

IN the last number of the Register, we made some remarks upon the duties of the Dentist in particular cases, and circumstances. We now propose some farther remarks upon this subject, but in reference to other particulars. We shall now consider Dental Medicine and Surgery, in other respects than the ordinary operations, such as filling, extracting, &c. Heretofore, the knowledge of pathological conditions, beyond the immediate tissues of the teeth, was very limited indeed, with the greater portion of the dental profession, and the consequence was that their treatment for these conditions was wholly at random and consequently inefficient, or was not attempted at all, the latter, with those sensible of deficiency would generally be the case. Some seem to suppose that if they have a few general ideas of the diseases of the teeth alone, that they are fully competent to treat them for those diseases. A proper consideration of the matter will convince any one of the fallacy of such a conclusion. No one is competent to treat any pathological condition without a thorough knowledge of that condition itself, at least so far as it may be known, and of all the circumstances that may modify or affect it. The treatment of any disease must be modified and governed by the attending peculiarities; and if these peculiarities are ignored the treatment is necessarily empirical, and usually inefficient. The diseases of the teeth—caries, for instance—may be influenced by a great variety of circumstances; such as a diminution of the systemic vitality, a diseased condition either general or local. Now, for the proper treatment of the dental tissues alone, it is all-important that the dentist be able to detect any diseases there may exist in the system, their nature and extent, and the influence, either direct or remote, that they may exert upon the teeth, without this, all the labor for the restoration and preservation of the teeth may be lost. With this view of the subject it is the duty of the dentist to extend his investigation into the cause, nature, and influence of disease as far as possible. No one, with a proper appreciation of the subject, will be satisfied with merely superficial attainments.

It is the business of the dentist to determine what influences are operating in producing caries of the teeth in any case; whether they are affected only by extraneous causes, or whether by vitiated secretions, or by a depraved condition of the general system, and if by the latter, what the peculiarities are. Not only should there be the ability to detect and diagnose disease, but also a knowledge of the proper treatment, especially that treatment which the welfare of the teeth demands.

It is not expected that in all cases the dentist shall attempt to employ general treatment, but there are many cases in which he may and it is even his duty to do it. The dentist can not always with propriety refer his patient to the physician. Often the physician does not know the object to be aimed at so well as the dentist.

It may not be the business of the dentist to treat general or local disease, outside of his specialty, with a view to its complete eradication, but it *is*, so far as a diversion of their pernicious influence upon the teeth is concerned.

If a knowledge of disease and its treatment is important to the dentist who operates only upon the teeth, much more is it important to him who operates upon and treats the parts and tissues in the vicinity of teeth. It is the business and proper function of the true dentist to treat not only the dental tissues, but also the surrounding parts that may be affected by the teeth or may affect them. The neighboring parts may become seriously affected by diseased teeth, and require very special treatment; disease of the antrum may be referred to as an example; tumors of various characters aside from diseased teeth, these sometimes occur of a malignant character; are sometimes confined to the soft parts; at others, involve the osseous tissues. In order to treat cases of this kind properly, the operator should be able to determine a malignant from a non-malignant tumor, or growth.

It is the legitimate work of the dentist to treat all the diseases of the teeth, and of the neighboring parts so far as they influence the teeth or are influenced by them. This may seem rather too much of a stretch when compared with the ordinary practice. There is no such thing as a good knowledge of the disease of a particular organ without a knowledge of the organs that may be in connection, both in respect to their normal and abnormal conditions. As a general thing, dentists give no attention to diseases of the parts, even of those that materially affect the teeth. This arises from the very circumscribed sphere which they have marked for themselves, and from the fact that the people make no demands, and have no expectation, in this direction. It is, however, the duty of the dentist, to do something more than perform a few simple operations upon the teeth, in a manner indicating that he ignores almost entirely their organization and vitality, and that they are susceptible of disease, or may be the cause of it in other parts. The dentist should be able to recognize the peculiarities and predisposition of any constitution as readily as the general physician or surgeon. He should be able to recognize the manner in which the teeth are liable to be affected in any given case, and how other parts, when diseased, will affect the teeth or be affected by them. Then, the next step is to know the appropriate treatment, and to possess the ability to employ it.

